# Could Selenium Administration Alleviate the Disturbances of Blood Parameters Caused by Lithium Administration in Rats?

**DOI:** 10.1007/s12011-014-9952-4

**Published:** 2014-03-28

**Authors:** Małgorzata Kiełczykowska, Joanna Kocot, Jacek Kurzepa, Anna Lewandowska, Renata Żelazowska, Irena Musik

**Affiliations:** Chair and Department of Medical Chemistry, Medical University of Lublin, Chodźki 4a, 20-093 Lublin, Poland

**Keywords:** Lithium, Selenium, Blood biochemistry, Morphology, Oxidative balance, Rats

## Abstract

Lithium is widely used in medicine, but its administration can cause numerous side effects. The present study aimed at the evaluation of the possible application of selenium, an essential and antioxidant element, as a protective agent against lithium toxicity. The experiment was performed on four groups of Wistar rats: I (control)—treated with saline, II (Li)—treated with lithium (Li_2_CO_3_), III (Se)—treated with selenium (Na_2_SeO_3_) and IV (Li + Se)—treated with lithium and selenium (Li_2_CO_3_ and Na_2_SeO_3_) in the form of water solutions by stomach tube for 6 weeks. The following biochemical parameters were measured: concentrations of sodium, potassium, calcium, magnesium, phosphorus, iron, urea, creatinine, cholesterol, glucose, total protein and albumin and activities of alkaline phosphatase, aspartate aminotransferase and alanine aminotransferase in serum as well as whole blood superoxide dismutase and glutathione peroxidase. Morphological parameters such as red blood cells, haemoglobin, haematocrit, mean corpuscular volume, mean corpuscular haemoglobin, mean corpuscular haemoglobin concentration, platelets, white blood cells, neutrophils as well as lymphocytes were determined. Lithium significantly increased serum calcium and glucose (2.65 ± 0.17 vs. 2.43 ± 0.11; 162 ± 31 vs. 121 ± 14, respectively), whereas magnesium and albumin were decreased (1.05 ± 0.08 vs. 1.21 ± 0.15; 3.85. ± 0.12 vs. 4.02 ± 0.08, respectively). Selenium given with lithium restored these parameters to values similar to those observed in the control (Ca—2.49 ± 0.08, glucose—113 ± 26, Mg—1.28 ± 0.09, albumin—4.07 ± 0.11). Se alone or co-administered with Li significantly increased aspartate aminotransferase and glutathione peroxidase. The obtained outcomes let us suggest that the continuation of research on the application of selenium as an adjuvant in lithium therapy seems warranted.

## Introduction

Lithium, recently included into the essential elements [1], has been found to influence numerous metabolic processes and exert divergent effects—both positive and negative. It is widely used in medicine, mostly in psychiatry as a mood stabilizer in the therapy of bipolar disorders [2]. However, the application of its compounds has also been studied in other fields of medicine, as an adjuvant in patients with thyroid diseases undergoing radioiodine therapy [3] and in the cure of neurodegenerative disorders [4]. As lithium displays beneficial action only within a strongly determined range, the therapy must be applied taking the appropriate precautions, all the more that no correlation between the dose and serum concentration of lithium occurs [5]. An overrun of the safe threshold can cause side effects including disturbances of the nervous and alimentary systems as well as renal and thyroid disorders [5–7]. Relationships between carbohydrate and lipid metabolism and lithium treatment have also been displayed [6]. Due to these facts, it has been recommended that not only lithium serum level but many other factors, e.g. creatinine and renal and thyroid functions, should be monitored during the period of therapy [8]. Furthermore, lithium administration has been revealed to affect oxidant balance [9, 10]. Our previous studies concerning the influence of lithium administration in drinking water have also shown that this element caused depletion in the antioxidant enzymes’ activities in some tissues of rats [11]. The attempts towards the possible application of essential antioxidant metals as protective agents against lithium action have already been undertaken [12, 13].

Selenium, an essential trace element, is necessary to correct an organism’s functions [14, 15]. Being a constituent of one of the main antioxidative enzymes—glutathione peroxidase—selenium is considered to be an antioxidant [14]. Research has indicated that bipolar and neurological disorders like epilepsy are associated with oxidative stress, and selenium compounds can display neuroprotective effects by different mechanisms, with the involvement of selenoproteins [14–19]. Selenium supplementation has been suggested to be beneficial in psychiatric disorders [20]. Furthermore, selenium has already been studied in regard to its possible application as a protective agent against toxicity caused by toxic metals and compounds [15, 21].

The estimation of the beneficial influence of selenium on organisms undergoing lithium administration is worth studying all the more that the possibility of the supplementation of drinking water with lithium is considered. It is caused by the fact that inverse associations between lithium level in drinking water and crime and suicide rates have been reported, although the results of the performed studies remain divergent [22]. It seems to be possible that selenium co-administration could exert beneficial effects in patients suffering from psychiatric or neurological disorders and undergoing lithium therapy. Aiming at verifying the presented assumption, we undertook this pilotage study—evaluation of the influence of selenium given in the form of acknowledged inorganic supplement sodium selenite, still used in clinical practice [23] and as a supplement of animal food [24], on blood parameters in rats receiving lithium.

## Materials and Methods

### Animals

The experiment was performed on male Wistar rats (32 animals, 130–160 g body mass, 6 weeks old). After an acclimatization period of 3 days, the animals were randomly divided into four groups (eight animals each): group I (control)—treated with saline, group II (Li)—treated with lithium (as Li_2_CO_3_) at a dose of 2.7 mg Li/kg b.w., group III (Se)—treated with selenium (as Na_2_SeO_3_) at a dose of 0.5 mg Se/kg b.w., and group IV (Se + Li)—treated simultaneously with lithium and selenium (Li_2_CO_3_ at a dose of 2.7 mg Li/kg b.w. and Na_2_SeO_3_ at a dose of 0.5 mg Se/kg b.w.). The doses and period of treatment were established basing on our previous studies regarding the effects of lithium and selenium on animal organisms to enable the comparison of the obtained results [11, 25]. The administered compounds were given in the form of water solutions. The administration was performed by stomach tube for a period of 6 weeks, once a day. The body mass of each animal was measured every day before administration, and the appropriate amount of selenium and/or lithium solutions was calculated. Rats had free access to standard feed (LSM produced by AGROPOL S.J., Motycz, Poland, without lithium and selenium supplementation) and drinking water. The study was performed according to statutory bioethical standards and approved by the I Local Ethical Commission of the Medical University of Lublin, acceptance no. 1/2013.

### Serum Biochemistry

After the end of the experiment, animals were sacrificed under thiopental narcosis, and samples of blood were collected. Electrolytes (sodium, potassium), as well as other biochemical parameters (calcium, magnesium, phosphorus, iron, urea, creatinine, cholesterol, glucose, total protein, albumin and activities of chosen blood enzymes: alkaline phosphatase (ALP), aspartate aminotransferase (AST), alanine aminotransferase (ALT)), were measured in serum using the AVL 9180 Electrolyte Analyzer (Roche, Germany) and Chemistry Analyzer BS-130 (Mindray, China), respectively.

### Assay of the Morphological Parameters

Morphological parameters: red blood cells (RBC), haemoglobin (HGB), haematocrit (HCT), mean corpuscular volume (MCV), mean corpuscular haemoglobin (MCH), mean corpuscular haemoglobin concentration (MCHC), platelets (PLT), white blood cells (WBC), neutrophils as well as lymphocytes were determined using the Scill Vet abc Plus + (HORIBA Medical, Germany) apparatus.

### Assay of Antioxidant Enzymes

Activities of antioxidant enzymes, superoxide dismutase (SOD) and glutathione peroxidase (GPx), were determined using diagnostic kits RANSOD and RANSEL, respectively, produced by RANDOX, and expressed in units per gram of HGB. The assays were performed using spectrophotometer SPECORD M40 (Zeiss, Jena, Germany).

### Statistical Analyses

The differences among the studied group were determined using a one-way analysis of variance (ANOVA), followed by the Tukey-Kramer test post hoc when appropriate. Software STATISTICA 6.0 was used. Values were considered significant with *p* < 0.05.

## Results

### Electrolytes and Biochemical Parameters

Neither lithium nor selenium alone altered sodium and potassium in serum. Co-administration of these two elements also exerted no effect. On the contrary, calcium and magnesium levels were affected (ANOVA *p* = 0.0142, *p* = 0.0054, respectively). Calcium was significantly increased vs. control in Li-treated animals, whereas Li and Se together restored Ca to the value comparable to that observed in the control group. Magnesium was markedly decreased in the Li group, whereas neither selenium alone nor lithium + selenium changed Mg level vs. the control. Selenium given with Li markedly increased Mg when compared to the Li-alone group. Phosphorus and iron were not changed when compared to the control in any studied group. Urea, creatinine, cholesterol and total protein in serum were not altered in comparison with the control group. Glucose and albumin levels were affected (ANOVA *p* = 0.0159, *p* = 0.0049, respectively). Glucose was significantly enhanced in Li-receiving rats vs. the control, and co-administration of Se decreased it markedly in comparison to the Li-alone group. Albumin level was decreased in Li-receiving animals, whereas neither Se alone nor Se given with lithium showed any effect vs. the control. However, in the group receiving Li + Se, albumin was significantly enhanced vs. the Li-alone group. All the reported results are presented below (see Table 1).

**Table 1:** The influence of lithium or/and selenium on electrolytes and biochemical parameters in serum of rats

Parameter	Control (*n* = 8)	Li + Se (*n* = 8)	Li (*n* = 8)	Se (*n* = 8)
Na [mmol/L]	142 ± 1	144 ± 2	143 ± 1	143 ± 4
K [mmol/L]	5.3 ± 0.4	5.2 ± 0.5	5.1 ± 0.2	5.1 ± 0.5
Ca [mmol/L]	2.43 ± 0.11	2.49 ± 0.08	2.65 ± 0.17***	2.56 ± 0.06
Mg [mmol/L]	1.21 ± 0.15	1.28 ± 0.09^*^	1.05 ± 0.08***	1.20 ± 0.03
P [mmol/L]	2.79 ± 0.41	2.98 ± 0.23	2.81 ± 0.04	2.80 ± 0.09
Fe [μmol/L]	25.4 ± 6.3	22.9 ± 3.9	20.8 ± 6.4	22.3 ± 4.7
Urea [mg/dL]	35.7 ± 4.0	35.8 ± 7.9	37.8 ± 7.8	35.8 ± 5.2
Creatinine [mg/dL]	0.34 ± 0.12	0.39 ± 0.05	0.34 ± 0.12	0.35 ± 0.02
Cholesterol [mg/dL]	50 ± 7	47 ± 7	46 ± 10	48 ± 3
Glucose [mg/dL]	121 ± 14	113 ± 26^**^	162 ± 31***	131 ± 36
Total protein [g/dL]	5.93 ± 0.22	6.17 ± 0.22	6.12 ± 0.28	6.09 ± 0.09
Albumin [g/dL]	4.027 ± 0.08	4.07 ± 0.11^*^	3.85 ± 0.12***	3.94 ± 0.08

Values are mean ± SD

*n* number of animals in the group

*p < 0.01, significantly different vs. Li group; **p < 0.05, significantly different vs. Li group; ***p < 0.05, significantly different vs. control group

### Morphological Parameters

Morphological parameters—red blood cells, haematocrit, haemoglobin, mean corpuscular volume, mean corpuscular haemoglobin, mean corpuscular haemoglobin concentration, platelets, white blood cells, neutrophils as well as lymphocytes were not altered by the administration of lithium or/and selenium in comparison with the control group (see Table 2).

**Table 2:** The influence of the administration of lithium or/and selenium on morphological parameters in rats

Parameter	Control (*n* = 8)	Li + Se (*n* = 8)	Li (*n* = 8)	Se (*n* = 8)
RBC [10^6^/mm^3^]	7.5 ± 0.6	7.8 ± 0.6	7.9 ± 0.3	7.8 ± 0.5
HGB [g/dL]	14.5 ± 0.3	14.3 ± 0.5	14.5 ± 0.4	14.7 ± 0.6
HCT [%]	42.4 ± 2.2	41.8 ± 2.7	43.2 ± 1.1	43.0 ± 2.8
MCV [μm^3^]	57 ± 3	54 ± 2	54 ± 2	55 ± 1
MCH [pg]	19.4 ± 1.5	18.7 ± 1.0	18.3 ± 0.6	19.0 ± 0.8
MCHC [g/dL]	34.0 ± 0.4	34.2 ± 1.3	33.6 ± 0.5	34.3 ± 1.1
PLT [10^3^/mm^3^]	715 ± 75	749 ± 92	710 ± 54	644 ± 78
WBC [10^3^/mm^3^]	4.2 ± 1.3	4.9 ± 2.7	4.2 ± 1.9	4.9 ± 1.6
Neutrophils [10^3^/mm^3^]	1.3 ± 0.6	1.1 ± 0.3	0.8 ± 0.3	0.9 ± 0.6
Lymphocytes [10^3^/mm^3^]	2.8 ± 0.9	3.7 ± 2.7	3.3 ± 1.6	3.7 ± 1.3

Values are mean ± SD

*n* number of animals in the group, *RBC* red blood cells, *HGB* haemoglobin, *HCT* haematocrit, *MCV* mean corpuscular volume, *MCH* mean corpuscular haemoglobin; *MCHC* mean corpuscular haemoglobin concentration, *PLT* platelets, *WBC* white blood cells

### Blood Enzymes

Statistical analysis showed the differences among the studied groups in the case of AST, ALT and GPx (ANOVA *p* = 0.0173, *p* = 0.0199, *p* < 0.0001, respectively). AST was increased significantly in the Se and Li + Se groups vs. the control. ALT in the Se-treated group was increased when compared to the control (see Table 3).

**Table 3:** The influence of the administration of lithium or/and selenium on blood enzymes

Parameter	Control (*n* = 8)	Li + Se (*n* = 8)	Li (*n* = 8)	Se (*n* = 8)
ALP [U/L]	154 ± 41	147 ± 42	193 ± 60	138 ± 20
AST [U/L]	148 ± 25	181 ± 18*	184 ± 25	195 ± 32*
ALT [U/L]	52 ± 9	63 ± 11	56 ± 5	68 ± 9*

Values are mean ± SD

*ALP* alkaline phosphatase, *AST* aspartate aminotransferase, *ALT* alanine aminotransferase, *n* number of animals in the group

*p < 0.05, significantly different vs. the control group

**Fig. 1:**
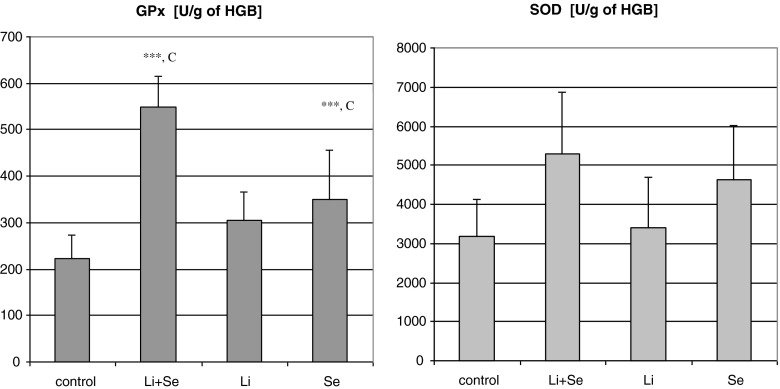
The influence of the administration of lithium or/and selenium on chosen whole blood antioxidant enzymes. *SOD* superoxide dismutase and *GPx* glutathione peroxidase. Values are mean ± SD, *n* = 8 (number of animals in the group). ***p < 0.001, significantly different vs. the control group, ^*c*^ *p* < 0.001 significantly different vs. the Li group

Whole blood GPx activity was enhanced in the Se and Li + Se groups vs. both control and Li-alone animals. Whole blood SOD was not changed in rats receiving lithium and/or selenium in comparison with the control (see Fig. 1).

## Discussion

In available data, there are rather few reports concerning correlations between lithium and selenium. Broberg et al. have found a negative correlation between lithium urinary concentration and plasma free thyroxine (T_4_) level as well as a positive correlation between urinary selenium and T_4_ in women who were residents of areas with high lithium level in drinking water [26]. Selenium deficiency and lithium administration have been found to be connected with autoimmune thyroid diseases [27].

In the current study, intragastric administration of lithium altered neither sodium nor potassium in serum. Similarly, no changes of plasma sodium in rats fed a Li-containing diet were reported [7].

Lithium administration disturbed the chosen biochemical parameters—calcium, magnesium, glucose and albumin in the serum of rats. Calcium was enhanced in Li-treated rats which is consistent with outcomes reported by Tandon et al. [28]. Selenium co-administration restored both magnesium and calcium levels in serum. The latter fact seems to be of great importance as hypercalcaemia was found to be associated with long-term use of lithium [29]. Moreover, an increased Ca/Mg ratio was found in the serum and cerebrospinal fluid of depressed subjects [30]. The current experiment also revealed an ameliorating effect of selenium in the case of glucose. It is a very important result considering the relationships between lithium and enzymes involved in glucose metabolism [29, 31, 32]. Intravenous lithium was reported to cause an increase in plasma glucose and depletion of plasma insulin in normal rats [33]. Studies also revealed the occurrence of hyperglycaemia as one of the metabolic disturbances noted in bipolar disorder patients [34]. In the present study, albumin was significantly decreased in Li-treated animals and restored by selenium administration, whereas no changes of total protein were shown. Tandon et al. did not observe any changes in albumin in the serum of rats fed diets with different protein levels, but the period of the experiment was a little shorter (1 month). Total protein was not disturbed [35].

This experiment did not reveal any Li effect on serum urea or creatinine. It could result from the fact that the Li dose was rather low. In rats fed a diet with added lithium, plasma urea was significantly depleted after 4 weeks, but creatinine remained unchanged in spite of the decreased creatinine clearance [7]. Another experiment performed on rats given a diet containing Li_2_CO_3_ showed that it was already after 7 days when a significant increase in serum urea as well as creatinine was observed, whereas urinary urea was strongly diminished pointing to an alteration of kidney functioning. Moreover, this effect remained throughout the experiment (28 days) [36]. Also, in mice receiving injections of lithium carbonate, the lowest dose did not alter serum creatinine, whereas higher ones caused its significant increase as early as after 14 days [37]. The current study showed no effect of lithium on urea and cholesterol. Ahmad et al. [6] reported the same outcomes concerning potassium in rats given lithium chloride, whereas urea was found to be enhanced and high density lipoproteins decreased. However, both the period of the experiment and the applied dose were greater. Glucose in turn was found to be increased both in this study and in the experiment performed by Ahmad et al. [6] which could show that glucose is a parameter more vulnerable to lithium’s presence in an organism and that lithium affects carbohydrate metabolism more quickly than that of lipids and nitrogen balance.

In the present study, serum ALP and AST were not changed markedly by Li treatment although slight enhancement was observed, whereas ALT remained unchanged. On the contrary, Ahmad et al. observed a significant decrease in all these enzymes’ activities in rats receiving lithium chloride in drinking water. However, the period of the experiment was a little longer [6].

In the present study, no applied treatment caused any changes of the morphological parameters. Similarly, dietary lithium given for a period of 2 months did not affect significantly haemoglobin and haematocrit in rats, but that longer time of exposure resulted in a well-marked increase in total leukocyte counts, neutrophils and lymphocytes [13]. Co-treatment of zinc restored total leukocyte counts and lymphocytes which could confirm the usefulness of antioxidants for alleviating lithium therapy side effects. The presented results show that longer administration of lithium can influence the immune system. As an adequate selenium level in an organism is connected with proper functioning of the immune system [38], these observations encourage further research on adjuvant selenium therapy in patients receiving lithium, particularly during long-term treatment.

Studies revealed that among other things, lithium toxicity can be connected with oxidative stress [6, 10, 13, 36], but contradicting outcomes were also reported [39, 40]. Furthermore, oxidative stress was also found to be involved into the pathophysiology of bipolar disorder [40, 41]. As long-term lithium therapy is used in the cure of this disease, the question of the influence of lithium on oxidative processes is an issue of great importance. The organisms developed a complex system of defence against oxidative stress which includes numerous substances, among other things antioxidant enzymes, namely superoxide dismutase, glutathione peroxidase and catalase [14, 40, 42, 43]. In this pilotage study, we investigated two of them: superoxide dismutase and glutathione peroxidase.

The period of the current experiment was very short when compared to psychiatric patients, and no lithium influence on SOD was displayed. However, even in such a short period, the tendency towards beneficial effect of selenium was shown. Lithium given for a longer period (2 months) caused a significant decrease in SOD in rats [13]. Khairova et al. [39] found decreased SOD in healthy subjects undergoing Li treatment. These outcomes seem to confirm our assumption regarding possible adjuvant application of any antioxidant in lithium treatment. In the present study, selenium markedly enhanced GPx vs. the control, whereas in the case of SOD, only a slight, insignificant increase was observed. Similar observations were reported by El-Demerdash and Nasr [21].

In available data, there are not too many reports regarding relationships between lithium and iron. However, the study concerning the levels of different elements in hair of haemodialysis patients revealed that the statistically significant increase in iron was accompanied with a slight decrease in lithium [44]. In the present study, serum iron as well as mean corpuscular haemoglobin was slightly decreased in the lithium-treated group, and selenium co-administration seemed to exert a slight beneficial effect. Dhawan et al. also observed depressed iron in lithium-administered rats [45]. As the period of our experiment was rather short, further studies could allow the clarification of the issue on whether lithium administration could disturb iron homeostasis and whether selenium might alleviate such effect.

Co-administration of selenium restored significantly the lithium-disturbed levels of calcium, magnesium, albumin and glucose, and a similar effect, although insignificant, was noted in the case of iron level. Antioxidant enzymes were enhanced. To conclude, the obtained outcomes of this pilotage experiment let us suggest that the application of selenium as an adjuvant in lithium therapy is worth considering. Further studies are needed to solve the issue of the proper dose and form of selenium supplementation.

## References

[1] Schöpfer J, Schrauzer GN (2011). Lithium and other elements in scalp hair of residents of Tokyo Prefecture as investigational predictors of suicide risk. Biol Trace Elem Res.

[2] Rybakowski JK, Suwalska A (2010). Excellent lithium responders have normal cognitive functions and plasma BDNF levels. Int J Neuropsychopharmacol.

[3] Bogazzi F, Giovannetti C, Fessehatsion R (2010). Impact of lithium on efficacy of radioactive iodine therapy for Graves’ disease: a cohort study on cure rate, time to cure, and frequency of increased serum thyroxine after antithyroid drug withdrawal. J Clin Endocrinol Metab.

[4] Luo J (2010). Lithium-mediated protection against ethanol neurotoxicity. Front Neurosci.

[5] Ng YW, Tiu SC, Choi KL (2006). Use of lithium in the treatment of thyrotoxicosis. Hong Kong Med J.

[6] Ahmad M, Elnakady Y, Farooq M, Wadaan M (2011). Lithium induced toxicity in rats: blood serum chemistry, antioxidative enzymes in red blood cells and histopathological studies. Biol Pharm Bull.

[7] Kortenoeven ML, Li Y, Shaw S (2009). Amiloride blocks lithium entry through the sodium channel thereby attenuating the resultant nephrogenic diabetes insipidus. Kidney Int.

[8] Collins N, Barnes TRE, Shingleton-Smith A, Gerrett D, Paton C (2010). Standards of lithium monitoring in mental health trusts in the UK. BMC Psychiatry.

[9] Bhalla P, Chadha VD, Dhar R, Dhawan DK (2007). Neuroprotective effects of zinc on antioxidant defense system in lithium treated rat brain. Indian J Exp Biol.

[10] Toplan S, Dariyerli N, Ozdemir S, Ozcelik D, Zengin EU, Akyolcu MC (2013). Lithium-induced hypothyroidism: oxidative stress and osmotic fragility status in rats. Biol Trace Elem Res.

[11] Kiełczykowska M, Musik I, Pasternak K (2008). Relationships between silicon content and glutathione peroxidase activity in tissues of rats receiving lithium in drinking water. Biometals.

[12] Chadha VD, Bhalla P, Dhawan DK (2008). Zinc modulates lithium-induced hepatotoxicity in rats. Liver Int.

[13] Malhotra A, Dhawan DK (2008). Zinc improves antioxidative enzymes in red blood cells and hematology in lithium-treated rats. Nutr Res.

[14] de Freitas AS, Rocha JB (2011). Diphenyl diselenide and analogs are substrates of cerebral rat thioredoxin reductase: a pathway for their neuroprotective effects. Neurosci Lett.

[15] Glaser V, Moritz B, Schmitz A, Dafré AL, Nazari EM, Rauh Müller YM, Feksa L, Straliottoa MR, de Bem AF, Farina M, da Rocha JB, Latini A (2013). Protective effects of diphenyl diselenide in a mouse model of brain toxicity. Chem Biol Interact.

[16] Kim HK, Andreazza AC, Yeung PY, Isaacs-Trepanier C, Young LT (2014). Oxidation and nitration in dopaminergic areas of the prefrontal cortex from patients with bipolar disorder and schizophrenia. J Psychiatry Neurosci.

[17] Nazıroğlu M, Yürekli VA (2013). Effects of antiepileptic drugs on antioxidant and oxidant molecular pathways: focus on trace elements. Cell Mol Neurobiol.

[18] Yürekli VA, Nazıroğlu M (2013). Selenium and topiramate attenuates blood oxidative toxicity in patients with epilepsy: a clinical pilot study. Biol Trace Elem Res.

[19] Rosa AR, Singh N, Whitaker E, de Brito M, Lewis AM, Vieta E, Churchill GC, Geddes JR, Goodwin GM (2014). Altered plasma glutathione levels in bipolar disorder indicates higher oxidative stress; a possible risk factor for illness onset despite normal brain-derived neurotrophic factor (BDNF) levels. Psychol Med.

[20] Benton D (2002). Selenium intake, mood and other aspects of psychological functioning. Nutr Neurosci.

[21] El-Demerdash FM, Nasr HM (2013). Antioxidant effect of selenium on lipid peroxidation, hyperlipidemia and biochemical parameters in rats exposed to diazinon. J Trace Elem Med Biol.

[22] Giotakos O, Nisianakis P, Tsouvelas G, Giakalou VV (2013). Lithium in the public water supply and suicide mortality in Greece. Biol Trace Elem Res.

[23] Savory LA, Kerr CJ, Whiting P, Finer N, McEneny J, Ashton T (2012). Selenium supplementation and exercise: effect on oxidant stress in overweight adults. Obesity (Silver Spring).

[24] Pavlović Z, Miletić I, Jokić Ž, Pavlovski Z, Škrbić Z, Šobajić S (2010). The effect of level and source of dietary selenium supplementation on eggshell quality. Biol Trace Elem Res.

[25] Musik I, Kiełczykowska M, Kocot J (2013). Oxidant balance in brain of rats receiving different compounds of selenium. Biometals.

[26] Broberg K, Concha G, Engström K, Lindvall M, Grandér M, Vahter M (2011). Lithium in drinking water and thyroid function. Environ Health Perspect.

[27] Prummel MF, Strieder T, Wiersinga WM (2004). The environment and autoimmune thyroid diseases. Eur J Endocrinol.

[28] Tandon A, Nagpaul JP, Bandhu H, Singh N, Dhawan DK (1999). Effect of lithium on hepatic and serum elemental status under different dietary protein regimens. Biol Trace Elem Res.

[29] Giusti CF, Amorim SR, Guerra RA, Portes ES (2012). Endocrine disturbances related to the use of lithium. Arq Bras Endocrinol Metabol.

[30] Levine J, Stein D, Rapoport A, Kurtzman L (1999). High serum and cerebrospinal fluid Ca/Mg ratio in recently hospitalized acutely depressed patients. Neuropsychobiology.

[31] Csutora P, Karsai A, Nagy T (2006). Lithium induces phosphoglucomutase activity in various tissues of rats and in bipolar patients. Int J Neuropsychopharmacol.

[32] Wiernsperger N, Rapin JR (2010). Trace elements in glucometabolic disorders: an update. Diabetol Metab Syndr.

[33] García Hermida O, Fontela T, Ghiglione M, Uttenthal LO (1994). Effect of lithium on plasma glucose, insulin and glucagon in normal and streptozotocin-diabetic rats: role of glucagon in the hyperglycaemic response. Br J Pharmacol.

[34] Chang HH, Chou CH, Chen PS (2009). High prevalence of metabolic disturbances in patients with bipolar disorder in Taiwan. J Affect Disord.

[35] Tandon A, Dhawan DK, Nagpaul JP (1998). Effect of lithium on hepatic lipid peroxidation and antioxidative enzymes under different dietary protein regimens. J Appl Toxicol.

[36] Nciri R, Allagui MS, Bourogaa E (2012). Lipid peroxidation, antioxidant activities and stress protein (HSP72/73, GRP94) expression in kidney and liver of rats under lithium treatment. J Physiol Biochem.

[37] Nciri R, Allagui MS, Croute F, Vincent C, Elfeki A (2008). Effects of low doses of Li carbonate injected into mice. Functional changes in kidney seem to be related to the oxidative status. C R Biol.

[38] Verma S, Hoffmann FW, Kumar M (2011). Selenoprotein K knockout mice exhibit deficient calcium flux in immune cells and impaired immune responses. J Immunol.

[39] Khairova R, Pawar R, Salvadore G (2012). Effects of lithium on oxidative stress parameters in healthy subjects. Mol Med Rep.

[40] Machado-Vieira R, Andreazza AC, Viale CI (2007). Oxidative stress parameters in unmedicated and treated bipolar subjects during initial manic episode: a possible role for lithium antioxidant effects. Neurosci Lett.

[41] Brüning CA, Prigol M, Luchese C, Pinton S, Nogueira CW (2012). Diphenyl diselenide ameliorates behavioral and oxidative parameters in an animal model of mania induced by ouabain. Prog Neuropsychopharmacol Biol Psychiatry.

[42] Naziroğlu M (2007). New molecular mechanisms on the activation of TRPM2 channels by oxidative stress and ADP-ribose. Neurochem Res.

[43] Nazıroğlu M (2012). Molecular role of catalase on oxidative stress-induced Ca(2+) signaling and TRP cation channel activation in nervous system. J Recept Signal Transduct Res.

[44] Ochi A, Ishimura E, Tsujimoto Y (2011). Trace elements in the hair of hemodialysis patients. Biol Trace Elem Res.

[45] Dhawan D, Singh A, Singh B, Bandhu HK, Chand B, Singh N (1999). Effect of lithium augmentation on the trace elemental profile in diabetic rats. Biometals.

